# “Don’t Let Medicines Go to Waste”—A Survey-Based Cross-Sectional Study of Pharmacists’ Waste-Reducing Activities Across Gulf Cooperation Council Countries

**DOI:** 10.3389/fphar.2020.01334

**Published:** 2020-08-28

**Authors:** Faten Alhomoud

**Affiliations:** Department of Pharmacy Practice, College of Clinical Pharmacy, Imam Abdulrahman Bin Faisal University, Dammam, Saudi Arabia

**Keywords:** medication wastage, drug waste, unused medicine, unwanted medicine, medication disposal, medication non-adherence, cross-sectional survey, Gulf Cooperation Council countries

## Abstract

**Objectives:**

Medication waste has a negative environmental and economic impact. From avoiding unnecessary supply to recycling medicines that are no longer needed, pharmacists are placed in an advantageous position to minimise medication waste. Thus, the aim of this study was to detect waste-minimising activities undertaken by pharmacists to limit medication waste and to evaluate their importance for medication waste minimisation and feasibility for implementation.

**Methods:**

A cross-sectional survey of 277 participants, conveniently selected from six Gulf countries. The eligibility criteria were pharmacists or pharmacy technicians, Arabic- or English-speaking individuals, aged ≥18 years, and living in the Gulf countries. An online self-administered survey was distributed during December 2019 and February 2020, *via* e-mail and social networks; it included the purpose of the study with a link to the webpage that hosted the questionnaire. Data were collected through the online survey site QuestionPro.com. All analyses were performed in SPSS Version 23 and Microsoft Excel.

**Results:**

The majority of participants were female (175/277; 63%), hospital pharmacists (206/277; 74%), and had more than 10 years of work experience (100/277; 36%). This study indicates that most of the suggested activities (14/21) were implemented by the majority of pharmacists for minimising medication waste, except four activities in the dispensing stage and three activities in the leftover stage. Activities performed at the prescribing, dispensing and leftover stages were considered very important or important for reducing medication waste and very feasible or feasible for implementation in practice.

**Conclusions:**

Many waste-minimising activities were undertaken by pharmacists in the prescribing, dispensing, and leftover stages. However, although waste-minimising activities were perceived as important for reducing waste and feasible for implementation in practice, not all activities were implemented in daily practice.

## Introduction

In terms of prescription medications, medication waste is defined as any medication that remains unused or expires anywhere along the medicine supply chain (West et al., 2015). Medication waste can occur at the prescriber, pharmacist and patient levels. For example, unnecessary drugs or excessive quantities may be prescribed by the physician (prescribing stage). Medicines on repeat prescriptions may be dispensed without proper review, or dispensed in large quantities as the manufacturer’s package size may exceed the amount required for treatment (dispensing stage). Patients may intentionally (e.g., change in belief, unsatisfactory effect, side effects) or unintentionally (e.g., forgetfulness) fail to take medications as prescribed, which could result in medication remaining unused. They may also habitually order every medicine on a repeat prescription regardless of the need, or may recover from a condition or have a change to a condition that necessitates a change in medication, and this may result in accumulation of unused medicines at home (Abahussain et al., 2012; West et al., 2015; Al-Shareef et al., 2016; Bekker et al., 2018b).

The majority of unwanted or expired medicines are disposed of *via* household waste, toilet, or sink and only the minority are returned to pharmacies in Gulf Cooperation Council (GCC) countries (Abahussain et al., 2012; Al-Shareef et al., 2016). This waste has an undesirable impact, both environmentally and economically. The contamination of water and food with pharmaceutical residues can harm living beings. In addition, this waste costs healthcare systems hundreds to millions of dollars every year due to, for example, unused drugs, disposal costs and time spent on activities such as prescribing and dispensing (Abahussain et al., 2012; Al-Shareef et al., 2016). Efforts to minimise the undesirable environmental and economic burden through medication waste prevention strategies are thus required.

Medication waste in GCC countries is expected to increase due to the fact that the population in the Gulf is anticipated to expand due to high levels of urbanisation and a strong expatriate presence (Alkhamis et al., 2014). In addition, the population of the GCC is ageing and as people get older they are more likely to develop ≥1 long-term conditions (LTCs) and take ≥1 regular medicines (Abou-Auda, 2003; SFDA, 2012; Chahine et al., 2013; WHO, 2015), and consequently this may escalate prescribing volumes.

Irrational use of medicines, extravagant prescribing or the sale of medicines without prescriptions in the community pharmacies, providing citizens with free medications, are also reasons that may contribute to medication waste (Abou-Auda, 2003; WHO, 2011; Chahine et al., 2013; Alkhamis et al., 2014). It is estimated that more than half of all medicines are improperly sold, prescribed, or dispensed (WHO, 2011). Additionally, approximately 50% of patients do not take their medications as prescribed (WHO, 2011).

The wastage problem is universal among developed countries. For example, annual drug wastage amounts to about £300 million in England, while consumers in the United States (US) wasted more than $418 billion in 2012 due to suboptimal use of medicines (Trueman et al., 2010; Express Scripts, 2013). A study found that the mean number (SD) of home medications found expired, unused, or deteriorated was 2.2 (2.7) and 2.7 (1.9) per household in Saudi Arabia and other Gulf countries respectively (Abou-Auda, 2003). The estimated medication waste was 25.8% in Saudi Arabia and 41.3% in other Gulf countries. A total of ~$150 million was spent by families in Saudi Arabia and other Gulf countries on medications that were never used (Abou-Auda, 2003).

As key players in the pharmaceutical supply chain, pharmacists are in a great position to minimise medication waste (FIP, 2015). They can reduce medications kept in stock, collect unused medications, educate patients about waste, and evaluate and limit medication being dispensed (Bekker et al., 2018b). However, only a limited number of studies have been conducted to identify activities implemented by pharmacists in routine practice to minimise medication waste in the GCC countries (West et al., 2014). Doing so could provide an insight into activities that are used to minimise medication wastage and encourage implementation of such activities in routine practice. Thus, this study aimed at identifying activities undertaken by pharmacists to minimise medication wastage and assessing the extent to which these activities are implemented, and their importance and feasibility for implementation.

## Methods

### Design

A descriptive, cross-sectional online survey was conducted during December 2019 and February 2020 throughout the GCC region.

### Study Population

The study population consisted of pharmacists or pharmacy technicians, Arabic- or English-speaking individuals, aged ≥18 years, and living in the Gulf countries. A convenience sampling method was used in this survey to attract respondents from a large geographical area and facilitate the recruitment of a large sample.

### Survey Distribution and Return

An internet-based survey questionnaire was distributed. To allow for secure and anonymous participation, the internet-based survey site QuestionPro hosted the online survey. All participants were informed throughout the survey website that the survey should take about 10 min to fill out and were asked to read the participant information sheet online. Informed consent was obtained from all participants before they completed the online survey. Confidentiality was maintained and participation was voluntary throughout the study. Only questionnaires with completed responses were included in the analysis.

### Participant Identification and Recruitment

A survey invitation e-mail was sent by the researcher through their university e-mail network to potential participants (i.e., pharmacists working in an academic setting in Gulf countries). The e-mail contained an introductory statement about the study with a hyperlink to the web-based survey. The e-mail offered transformation of the URLs into direct links to the QuestionPro website.

The researcher also sent WhatsApp^®^ messages to their contacts and professional colleagues working in different sectors across Gulf countries (i.e., academic, industrial, community and hospital sectors), to increase the number of responses. Distribution of the survey link and the project to as many colleagues and friends as possible (“snowball principle”) was requested.

The electronic survey was also posted on Twitter^®^ and Facebook^®^ to the following organisations’ pages: Saudi Society of Clinical Pharmacy, Saudi Pharmacists Society, Eastern Province Pharmacists Club, Bahrain Pharmacists Society, Kuwait Pharmaceutical Association, Oman Pharmaceutical Society, and Emirates Medical Association. The survey homepage provided a short description of the study’s aim, the estimated completion time, contact information of the researcher responsible for the study, and some short instructions on how to complete the survey.

To increase the recruitment rate, small cards which contained the introductory statement about the study and a quick response (QR^®^) survey code leading to the survey homepage were also distributed among pharmacists and pharmacy technicians who participated in the Saudi International Pharmaceutical Sciences Meetings and Workshops (SIPHA) 2020 held in Riyadh, Saudi Arabia, in January 2020.

### Survey Development and Description of Final Version

Bekker’s instrument (Bekker et al., 2018b) is a self-administered survey instrument that has been developed by researchers at the Department of Clinical Pharmacy, Division Laboratories and Pharmacy, University Medical Centre Utrecht, and has previously been used in the Netherlands. The original survey questionnaire included a total of 14 activities that pharmacists can undertake or implement to minimise medication waste. It consisted of four sections: the prescribing stage (two activities), the dispensing stage—pharmacy-related activities (five activities), the dispensing stage—patient-related activities (four activities), and the leftover stage (three activities).

For each activity, pharmacists/pharmacy technicians were asked to indicate whether the activity was implemented in their country (yes/no), to rank its importance in minimising medication waste and the feasibility of implementing it in practice. Answers for both the importance in minimising medication waste and the feasibility of implementing it in practice were measured on 5-point Likert scales with descriptive choices ranging from one, indicating the activity as “not important” or “not feasible”, to five, “very important” or “very feasible”. Pharmacists or pharmacy technicians were also able to add other activities if these were not included.

For the present study, the original questionnaire was reviewed, extended and modified by a group of pharmacists (i.e., one academic, one hospital, and one community pharmacist) for relevance, appropriateness and acceptability. The modified survey instrument included a total of 21 activities, 14 of which were drawn directly from Bekker’s questionnaire whilst seven were generated by a group of pharmacists. Of the 21 activities, two were in the prescribing stage, 11 were in the dispensing stage—pharmacy-related activities, four activities were in the dispensing stage—patient-related activities, and four were in the leftover stage. The modified questionnaire also requests some personal information such as age, gender, educational level, years of work experience, and work setting and their current country of residence. Three pharmacists who were well experienced in the subject of the questionnaire were then invited to review the modified questionnaire. They were asked to comment on the questions added to the original Becker’s instrument in terms of relevancy, clarity and comprehension. They were also invited to give their comments for improving the questionnaire and write down any new questions that they think should be added. No changes were made to the modified questionnaire.

Prior to data collection, the questionnaire was translated from English to Arabic in which two pharmacy researchers translated the questionnaire independently (parallel blind translation technique) (Behling and Law, 2000), then both versions were compared and any inconsistencies were resolved by discussion. The translated version was assessed by a further Arabic-speaking pharmacist for clarity and comprehension. Then, the questionnaire was pretested with three pharmacists and adjusted accordingly (the pilot sample was not included in the study sample).

### Sample Size

To calculate sample size for the estimate prevalence with 95% confidence interval and 5% margin of error, the sample size can be estimated using the following formula:

N=((Z1-β+Zα/2)2 P(1-P))/E2×DEFF

Where:

P = estimated prevalence or proportion of event of interest for the study (50%)
E = Desired precision (or margin of error) (e.g., 0.05 for ±5%)
Zα/2 = 1.96, Z value for α = 0.05 or 95% confidence interval
Z1-β = 0.84, Z value for β = 0.20 or power of 0.8

N = ((0.84 + 1.96)2 0.5(1-0.5))/0.05

N = 280

To find the final adjusted sample size, allowing for a non-response rate of 10% in the above example, the adjusted sample size will be 280/(1–0.10) = 280/0.90 = 311.

### Data Management and Analysis

Once the survey was closed, data were downloaded from the QuestionPro website and imported directly into Statistical Package for the Social Sciences (SPSS, IBM Corporation, Armonk, NY, USA, Version 23) and Microsoft Excel (2010) (Microsoft, Albuquerque, NM, USA) for analysis. The accuracy of the gathered data was assessed by visual inspection by two researchers who were independent of the study. As part of the survey design, a mandatory response to all questions was required.

Descriptive analyses were performed by using counts and frequency [n (%)] for categorical variables, and median and interquartile range (IQR) for continuous variables. The results were also presented in tabular and graph forms. In the analysis more than 50% of the pharmacists within a country should have reported implementing the activity in order to be counted to weight the frequency scores. This is because, if fewer than half of the pharmacists within a country reported conducting a certain activity, it was assumed to be performed randomly.

## Results

### Survey Response and Participants’ Characteristics

A total of 277 pharmacists or pharmacy technicians completed the questionnaire from six GCC countries. The majority of participants were female (175/277; 63%), and were aged 25–34 years (146/277; 53%). The majority held an undergraduate qualification (161/277; 58%), were hospital pharmacists (206/277; 74%), employed in the government sector (236/277; 85%) and had more than 10 years of work experience (100/277; 36%). The country where most of the participants were located was Saudi Arabia (159/277; 57%). The majority of participants (237/277; 85%) reported that the medicines whose wastage they most tried to reduce were unaffordable or inaccessible ones. Participants’ characteristics are shown in **Table 1**.

**Table 1:** Characteristics of participants recruited into the study (N = 277).

Parameter		N	%
Gender	Male	102	37
	Female	175	63
Age (years)	18–24	25	9
	25–34	146	53
	35–44	85	31
	45–54	18	6
	≥ 55	3	1
Education	Postgraduate	74	27
	Undergraduate	161	58
	Diploma	42	15
Occupation*	Hospital pharmacy	206	74
	Community pharmacy	19	7
	Academia	21	8
	Research centre	9	3
	Pharmaceutical company and industry	6	2
	Pharmacy drugstore	17	6
	Primary care clinic’s pharmacy	8	3
	Drug regulatory authority	5	2
Job sector	Government	236	85
	Private	41	15
Years of work experience	< 5 years	98	35
	5–10 years	79	29
	>10 years	100	36
Country of current residence	Saudi Arabia	159	57
	Kuwait	41	15
	Oman	35	13
	United Arab Emirates	18	6.5
	Bahrain	14	5
	Qatar	10	3.5
Type of medicine whose waste you try to reduce in your country	Unaffordable/inaccessible medicines	237	85
	Affordable/accessible	141	51

*Percentage adds up to more than 100% as participants could tick more than one option.

### Waste-Minimising Activities Taken by Pharmacists

#### The Prescribing Stage

**Figure 1:**
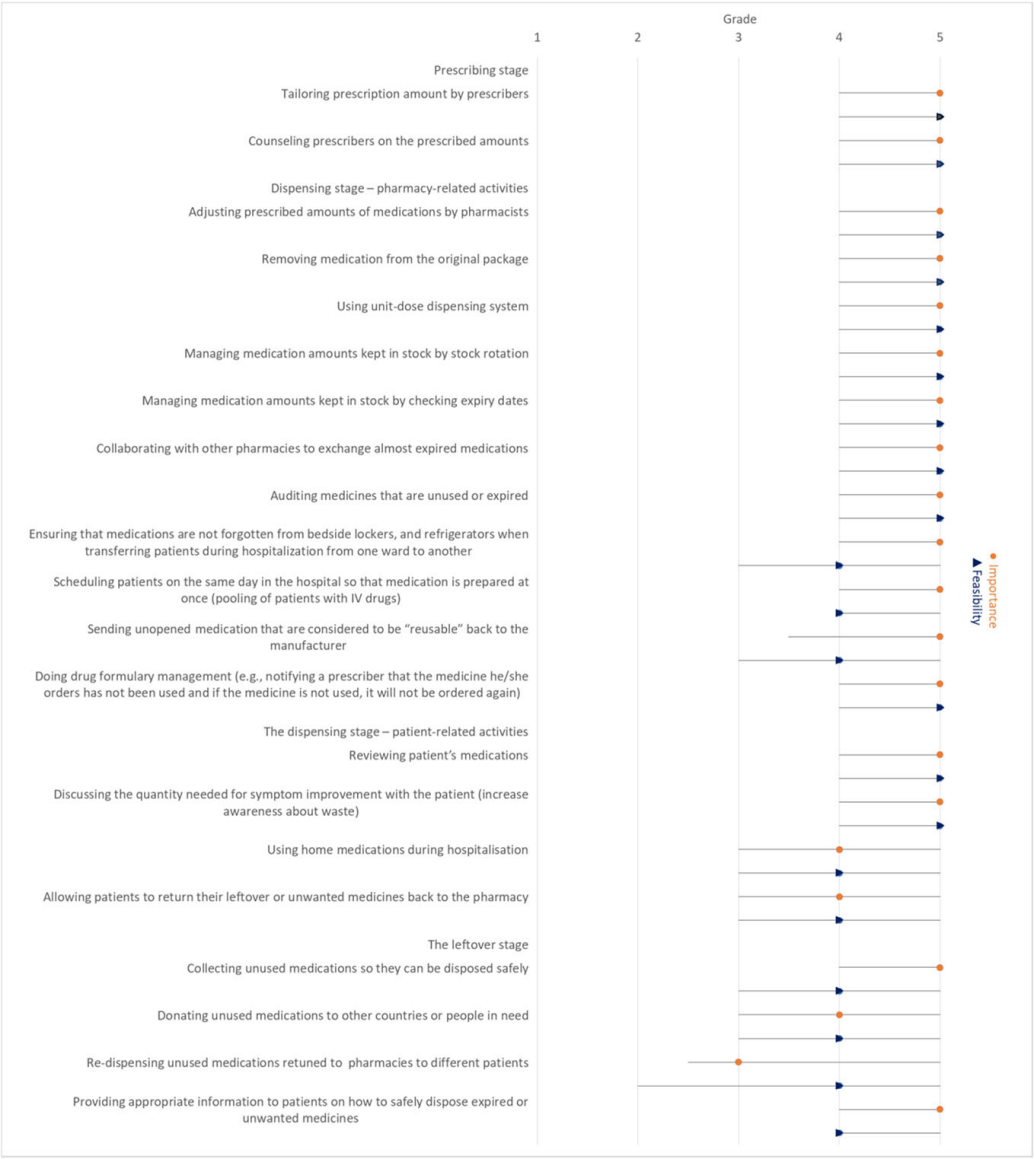
Median importance ranking to reduce medication waste for each activity and median feasibility ranking to implement the activity in practice (with interquartile range) reported by participants.

Of the responding countries, six reported tailoring prescription amount by prescribers and four reported counselling prescribers on the prescribed amounts (**Table 2**). These activities were considered very important for minimising medication waste (median 5) and very feasible to implement in practice (median 5) (**Figure 1**).

**Table 2:** Pharmacists’ activities to reduce mediation waste by countries.

Activity	Responses	Saudi Arabia (n =1 59) N (%)	Kuwait (n = 41) N (%)	United Arab Emirates (n = 18) N (%)	Qatar (n = 10) N (%)	Bahrain (n = 14) N (%)	Oman (n = 35) N (%)	Countries (n = 6) N
**Prescribing stage**								
Tailoring prescription amount by prescribers	Yes	144 (91)	31 (76)	17 (94)	10 (100)	11 (79)	30 (86)	6
	No	15 (9)	10 (24)	1 (6)	0	3 (21)	5 (14)	0
Counselling prescribers on the prescribed amounts	Yes	87 (55)	16 (39)	11 (61)	5 (50)	8 (57)	23 (66)	4
	No	72 (45)	25 (61)	7 (39)	5 (50)	6 (43)	12 (34)	2
**Dispensing stage—pharmacy-related activities**								
Adjusting prescribed amounts of medications by pharmacists	Yes	99 (62)	29 (71)	7 (39)	5 (50)	8 (57)	28 (80)	4
	No	60 (38)	12 (29)	11 (61)	5 (50)	6 (43)	7 (20)	2
Removing medication from the original package	Yes	137 (86)	38 (93)	11 (61)	6 (60)	13 (93)	35 (100)	6
	No	22 (14)	3 (7)	7 (39)	4 (40)	1 (7)	0	0
Using unit-dose dispensing system	Yes	140 (88)	40 (98)	13 (72)	8 (80)	12 (86)	32 (91)	6
	No	20 (12)	1 (2)	5 (27)	2 (20)	2 (14)	3 (9)	0
Managing medication amounts kept in stock by stock rotation	Yes	135 (85)	40 (98)	18 (100)	8 (80)	14 (100)	32 (91)	6
	No	24 (15)	1 (2)	0	2 (20)	0	3 (9)	0
Managing medication amounts kept in stock by checking expiry dates	Yes	151 (95)	39 (95)	18 (100)	10 (100)	14 (100)	34 (97)	6
	No	8 (5)	2 (5)	0	0	0	1 (3)	0
Collaborating with other pharmacies to exchange almost expired medications	Yes	111 (70)	33 (81)	13 (72)	7 (70)	10 (71)	30 (86)	6
	No	48 (30)	8 (19)	5 (28)	3 (30)	4 (29)	5 (14)	0
Auditing medicines that are unused or expired	Yes	136 (86)	34 (83)	18 (100)	9 (90)	13 (93)	32 (91)	6
	No	23 (14)	7 (17)	0	1 (10)	1 (7)	3 (9)	0
**Dispensing stage—pharmacy-related activities**								
Ensuring that medications are not left forgotten in bedside lockers and refrigerators when transferring patients during hospitalisation from one ward to another	Yes	60 (38)	12 (29)	11 (61)	7 (70)	6 (43)	8 (23)	2
	No	99 (62)	29 (71)	7 (39)	3 (30)	8 (57)	27 (77)	4
Scheduling patients on the same day in the hospital so that medication is prepared at once (pooling of patients with IV drugs)	Yes	102 (64)	13 (32)	8 (44)	6 (60)	4 (29)	13 (37)	2
	No	57 (36)	28 (68)	10 (56)	4 (40)	10 (71)	22 (63)	4
Sending unopened medications that are considered to be ‘reusable’ back to the manufacturer	Yes	37 (23.3)	9 (22)	7 (39)	1 (10)	2 (14.3)	6 (17)	0
	No	122 (76.7)	32 (78)	11 (61)	9 (90)	12 (85.7)	29 (83)	6
Doing drug formulary management (e.g., notifying a prescriber that the medicine he/she orders has not been used and if the medicine is not used, it will not be ordered again)	Yes	97 (61)	26 (63)	17 (94)	6 (60)	9 (64)	27 (77)	6
	No	62 (39)	15 (37)	1 (6)	4 (40)	5 (36)	8 (23)	0
**The dispensing stage—patient-related activities**								
Reviewing patient’s medications	Yes	122 (77)	28 (68)	13 (72)	9 (90)	11 (79)	30 (86)	6
	No	37 (23)	13 (32)	5 (28)	1 (10)	3 (21)	5 (14)	0
Discussing the quantity needed for symptom improvement with the patient (increase awareness about waste)	Yes	86 (54)	23 (56)	9 (50)	4 (40)	5 (36)	21 (60)	4
	No	73 (46)	18 (44)	9 (50)	6 (60)	9 (64)	14 (40)	2
Using home medications during hospitalisation	Yes	89 (56)	18 (44)	2 (11)	8 (80)	8 (57)	22 (63)	4
	No	70 (44)	23 (56)	16 (89)	2 (20)	6 (43)	13 (37)	2
**The dispensing stage—patient-related activities**								
Allowing patients to return their leftover or unwanted medicines to the pharmacy	Yes	69 (43)	16 (39)	4 (22)	8 (80)	9 (64)	32 (91)	3
	No	90 (57)	25 (61)	14 (78)	2 (20)	5 (36)	3 (9)	3
**The leftover stage**								
Collecting unused medications so they can be disposed of safely	Yes	105 (66)	23 (56)	10 (57)	10 (100)	11 (79)	30 (86)	6
	No	54 (34)	18 (44)	8 (44)	0	3 (21)	5 (14)	0
Donating unused medications to other countries or people in need	Yes	21 (13)	10 (24)	1 (6)	4 (40)	2 (14)	5 (14)	0
	No	138 (87)	31 (76)	17 (94)	6 (60)	12 (86)	30 (86)	6
Re-dispensing unused medications retuned to pharmacies to different patients	Yes	55 (35)	12 (29)	3 (17)	1 (10)	3 (21)	13 (37)	0
	No	104 (65)	29 (71)	15 (83)	9 (90)	11 (79)	22 (63)	6
Providing appropriate information to patients on how to safely dispose of expired or unwanted medicines	Yes	74 (47)	16 (39)	8 (44)	6 (60)	7 (50)	22 (63)	2
	No	85 (53)	25 (61)	10 (56)	4 (40)	7 (50)	13 (37)	4

#### Dispensing Stage—Pharmacy-Related Activities

Dispensing stage pharmacy-related activities’ were reported to be implemented by all the six GCC countries, except for the following activities: adjusting prescribed amounts of medications by pharmacists (4/6), ensuring that medications are not left forgotten in bedside lockers and refrigerators when transferring patients during hospitalisation from one ward to another (2/6), and scheduling patients with the same parenteral drug products on the same day in order to pool injection vials (2/6). Also, none of the counties reported sending unused/unopened/uncontaminated medications that are considered to be “reusable” back to the manufacturer (**Table 2**).

Overall, dispensing stage-pharmacy-related activities were considered very important for minimising waste (median ranking 5) and very feasible to implement in practice (median ranking 5), except for the following activities, which scored lower on feasibility for implementation (median ranking 4): ensuring that medications are not left forgotten in bedside lockers and refrigerators when transferring patients during hospitalisation from one ward to another, scheduling patients with the same parenteral drug products on the same day in order to pool injection vials, sending unused/unopened/uncontaminated medications that are considered to be ‘reusable’ back to the manufacturer (**Figure 1**).

#### Dispensing Stage—Patient-Related Activities

Of the responding countries, four reported discussing the quantity needed to improve a patient’s symptoms with the patient and using home medications during hospitalisation. Only three of the responding countries reported allowing patients to return their leftover or unwanted medicines to the pharmacy (**Table 2**). In the dispensing stage, patient-related activities were considered very important for waste minimisation (median ranking 5) and very feasible to implement in practice (median score 5), except for using home medications during hospitalisation and allowing patients to return their leftover or unwanted medicines to the pharmacy, which scored lower on importance for waste minimisation (median score 4) and feasibility for implementation (median score 4) (**Figure 1**).

#### The Leftover Stage

Only two of the countries provided appropriate information to patients on how to safely dispose of unwanted or expired medicines. None of the countries reported donating unused medications to other countries or people in need or re-dispensing unused medications retuned to pharmacies to different patients (**Table 2**). The activities in the leftover stage were considered very important to reduce medication wastage (median 5), except for donating unused medications to other countries or people in need and re-dispensing unused medications retuned to pharmacies to different patients, which were ranked as important (median 4) and neutral (median 3) respectively in terms of the importance in reducing medication waste. The activities in this stage were ranked feasible in terms of the feasibility of their implementation in practice (median 4) (**Figure 1**).

## Discussion

To the best of our knowledge, this is the first study to provide information on pharmacists’ activities to minimise medication wastage, their importance and feasibility in Gulf countries. In all stages of the pharmaceutical supply and use chain, pharmacists in the present study undertook various activities to minimise medication wastage. More than half of the participating countries revealed that they performed 14 of the suggested activities. The seven activities that were not conducted were: ensuring that medications are not forgotten in bedside lockers and refrigerators when transferring patients during hospitalisation from one ward to another, preparing medication at one time by scheduling patients with the same drug on the same day in the hospital (pooling of patients with IV drugs), sending unopened medications that are considered to be “reusable” back to the manufacturer, allowing patients to return their leftover or unwanted medicines to the pharmacy, donating unused medications to other countries or people in need, re-dispensing unused medications retuned to pharmacies to different patients, and providing appropriate information to patients on how to safely dispose of unwanted or expired medicines.

Pharmacists considered activities of the prescribing, dispensing, and leftover stages as either very important or important for reducing waste and very feasible or feasible for implementation in practice. However, re-dispensing unused medications retuned to pharmacies to different patients was ranked as “neutral” in terms of importance in minimising medication waste. This rating necessitates development of waste-reducing interventions to reduce medication waste.

To our knowledge, Saudi Arabia and other GCC countries do not have comprehensive medication waste management policies (Abou-Auda, 2003; Al-Shareef et al., 2016; Lucca et al., 2019). Under current regulations, people are not encouraged to return extra medications to the pharmacies. Pharmacies are not obligated by law to accept expired or unused medications from public (Abou-Auda, 2003; Al-Shareef et al., 2016; Lucca et al., 2019). Only few pharmacies are willing to take back unwanted medications for proper disposal but these medications that are returned unused to these pharmacies cannot be redistributed to other patients. So, they are treated as waste and subsequently destroyed. Many patients are not even aware of the presence of such service (Al-Shareef et al., 2016; Lucca et al., 2019). The World Health Organisation Guidelines for drug donation also state that sending medicines overseas that would not otherwise be used within the source country is unacceptable (WHO, 2010).

To date, there have been four main approaches to the problem of medicines waste (Jesson et al., 2005; Lucca et al., 2019). The first approach is thought patient education using health promotion leaflets, social networks, educational campaigns, television, and healthcare providers. The second is by improving prescribing practices to reduce waste. The third is through engagement of patients in their healthcare to improve their medication adherence. The fourth is through establishing a national return and medication disposal program in the community to cut waste.

As there are multiple causes of medication waste (Paterson and Anderson, 2002; Maeng et al., 2016; Bekker et al., 2018a; Bekker et al., 2018b; Bekker, 2018), no single intervention will sufficiently reduce the problem. To address the issue of medication waste, it would be prudent to base strategies on good prescribing, dispensing and medication-taking practices. Looking at the existing evidence of potential interventions, an example of a waste-minimising activity in the prescribing stage is to discuss sufficient medication quantities that meet patients’ needs with patients (Bekker et al., 2018a; Bekker et al., 2018b; Bekker, 2018), review prescribed medications periodically for each patient and discontinue unnecessary therapy (Bekker et al., 2018a; Bekker et al., 2018b; Bekker, 2018), dispense limited quantities of PRN medications to patients, prescribe new drugs for short duration (Bekker et al., 2018a; Bekker et al., 2018b; Bekker, 2018), provide expensive medications for 1-month supply (Bekker et al., 2018a; Bekker et al., 2018b; Bekker, 2018) and limit repeat prescription duration to 2 months (Maeng et al., 2016; Bekker et al., 2018a; Bekker et al., 2018b; Bekker, 2018). Various studies have suggested that the medication waste per prescription is lower when dispensing a 1-month supply compared to a 3-month supply (Domino et al., 2014; Doble et al., 2017; Miani et al., 2017).

Implementing shared decision-making between prescribers and patients (Baqir et al., 2017; Bekker, 2018) and increasing patient awareness about medication waste (Bekker et al., 2018b; Bekker, 2018) may also help in minimising medication waste. A study has shown that shared decision-making between prescribers and patients and medicine optimisation reviews can lead to a reduction in polypharmacy and tailor medicines to a patient’s individual needs and thus reduce medication waste arising from unnecessary medications (Baqir et al., 2017). Also, providing information on improving patients’ adherence to medicines and increasing awareness about medication waste that may occur due to non-adherence during consultation may help to tackle the problem.

Regarding the dispensing stage, transferring medications close to expiry to other pharmacies that are able to dispense them to patients in time may help in reducing medication waste (Baqir et al., 2017; Bekker, 2018). Pharmacies could request medications that are needed and not in stock using an online database and can register medications close to expiratory date on the database. Preparing liquids on a daily basis and scheduling patients that require parenteral preparations on the same day may help in preventing waste of medication prepared or compounded in the pharmacy (Fasola et al., 2014; Tilson et al., 2014). Splitting medication packages into smaller quantities, and using multidose vials and optimal vial sizes are all ways to minimise medication waste. Evaluating patients’ medications by conducting medication reviews to decrease the number of medications being unnecessarily dispensed to them, checking whether patients run out of stock before ordering a new refill, and understanding patients’ experiences and needs related to taking their medications could be other ways of waste-minimisation (Jesson et al., 2005; Bekker et al., 2018b).

Not only prescribers and pharmacists but also patients could play an important role in preventing medication waste. In Gulf countries, medications are provided to the citizens free of charge and many people still hold the idea that, when they visit a clinic or a hospital, they must be dispensed a prescription medication (Abou-Auda, 2003). The other reason for waste is that some prescription medicines can be purchased without prescription from community pharmacies in some Gulf countries (Abou-Auda, 2003). Thus, packs of drugs or the dosage label should be marked with their cost to remind the patients how much the treatment costs (Torjesen, 2015).

A well-run disposal and collection programme for proper collection and disposal of unused medicines is needed. Drug-take back programmes are not well established in all GCC countries (Abou-Auda, 2003; Abahussain et al., 2012). Thus, new laws or regulations which allow pharmacists to accept returned prescription medicines from consumers, indicate what medications might be recycled, and who might be eligible to return what medications should be implemented. One of the reasons that exacerbates the medication waste problem is the absence of guidelines on what to do with returned medicines from the public (Abou-Auda, 2003; Abahussain et al., 2012). Information on the types and quantities of returned, unused medicines from the public should be provided first before implementing the drug take-back programme.

A better understanding of the reasons leading to unused medicines in the first place is essential (Mackridge and Marriott, 2007). For example, is the reason for returning a medicine due to a change in the prescription, accumulated medicines or non-adherence due to adverse drug reactions or ineffective therapy? By knowing the reasons, pharmacists can educate patients about drug stockpiling, poor adherence, costs and waste of medications.

Installing regulations aimed at minimising medication waste through positive or negative enforcement and national awareness campaigns should be encouraged. For example, requiring healthcare providers to prescribe and dispense appropriate quantities of medication or providing financial support for disposal or rational prescribing and dispensing.

Collecting drugs after patients change medicines, stop medications, or die might be conducted, especially for tablets or capsules that are sealed in the manufacturer’s original, unopened, tamper-evident packaging and the pharmacists should examine these drugs for signs of tampering before releasing them for reuse, to ensure integrity of the product. Hospitals might also recycle unused medications returned from patients’ floors, wherever the original unit dose remains intact.

A study indicated that one-quarter of the unused or retuned medicines were suitable for re-use, with almost one-third of these being essential medicines per the World Health Organisation List (Mackridge and Marriott, 2007). However, a significant change to the medicine may occur as not all returned medicines have been stored in accordance with the manufacturer’s instructions. However, by utilising “newer” packaging technologies, including “temperature-sensitive smart” labels that react to humidity and temperature and tamper-evident seals, as well as current stability testing guidance, it would be possible to identify inappropriately stored medicines (Mackridge and Marriott, 2007). Thus, reuse of medicines may be considered by Gulf countries.

Under current regulations, even if some pharmacies collect unused medicines, these medicines cannot be supplied (or redistributed) to other patients (Abou-Auda, 2003). As such, they are treated as waste and subsequently destroyed due to commonly cited barriers of tampering and storage (Abou-Auda, 2003; Sejpal, 2007). It is believed that the safety and quality of returned medicines cannot be guaranteed as there is a potential for these medicines to be tampered with or stored in inappropriate conditions (Sejpal, 2007; Daughton et al., 2010; WHO, 2010). Thus, limiting the recycling programme to only new prescriptions, perhaps within 1 month of original issue can be a starting point. The consumers would have to return the medicine to the same place where it was issued, and fill out a form establishing that the medicine was properly stored and checking off the reason(s) why it was being returned.

Public education on the prompt return of unused medicines and proper storage of medications is needed to improve reusability of patients’ waste medicines. Alshareef et al. (Al-Shareef et al., 2016) found that there is a lack of information provided to patients in Saudi Arabia regarding correct disposal of unwanted medicines, a focus on other content during patient counselling, and workforce issues. Gulf countries should develop and sponsor drug collection programmes for collection and redistribution of unused medicines to patients who cannot afford them. Disposal bins could be provided in the supermarkets, GP surgeries to enable patients to return unused medicines to the pharmacy (Bekker et al., 2018b; Bekker, 2018). In addition, stickers or information leaflets placed on medication packages that request the return of unused medicines to the pharmacy is another way to overcome this problem. Raising patients’ awareness about environmental concerns associated with inappropriate disposal could be a motivator for returning medication to pharmacies (Tong et al., 2011). Changing people’s behaviours by providing small financial incentives for returning unused medication packages may reduce the environmental pollution and increase proper disposal. Waste medication could be recycled into similar products. The inner and outer packaging could be reprocessed, as could the active pharmaceutical ingredients of the medication.

Strengths: 1) to the best of our knowledge, this is the first multi-country study to provide information on pharmacist’s activity to minimise the medication wastage and identify the areas of potential improvement; 2) the short time the survey was performed (avoiding bias for long-term answers) and the large number of responses, which met the planned sample size represented a second strength of this study. For the current study, several limitations were detected: 1) this study applied only to pharmacists and pharmacy technicians—the reasons for the wastage related to the patients or prescribers, were not investigated; 2) the use of a convenience sample and self-administration of questionnaire could lead to responder bias; 3) only pharmacists and pharmacy technicians were involved, most were women and mean age was quite young; 4) since not all pharmacists and pharmacy technicians of the countries approached took part in the study, subjects may not be representative for their whole countries; 5) some activities might have been missed as not all pharmacists from a particular country responded to the survey; however, when we asked participants to report what other actions were taken to limit medication waste, no additional activities were identified.

## Conclusions

This study indicates that pharmacists in the six GCC countries have undertaken many activities to reduce medication waste, considering the prescribing, dispensing and leftover stages as important for reducing waste and feasible for implementation in practice. The re-dispensing of unused medicines returned to pharmacies was scored as “neutral”. None of the countries reported sending the unused/unopened/uncontaminated medication that are considered “reusable” back to the manufacturer. There are needed complex activities with multi-stakeholder involvement (including health authorities, manufacturers, distributors, prescribers, pharmacists, and patients), to reduce medication waste, such as: shared decision makers between prescribers and patients, transferring medication close to expiry to other pharmacies, redistribution to the unused medicines to other patients, correct disposal of unwanted medicines, package split, and ensure adherence to treatment.

Pharmacists, in particular, can play a significant role in preventing the damage that is occurred by medication waste, which is not only an economic waste but also contributes to water pollution. The survey was completed by a large number of pharmacists and identified many activities already implemented, but also several additional steps which may be used more frequently. More studies are warranted to precisely quantify drug waste and types of medication wasted in order to set targets for waste minimisation and reuse and recycling and cost reduction. No research has been undertaken in Gulf countries to understand patients’ and healthcare providers’ views on the redistribution of medicines. Thus, research on this topic is vital, especially if educational campaigns are to be designed to address concerns and negative views about medicine redistribution that may be held by the public.

## Data Availability Statement

The raw data supporting the conclusions of this article will be made available by the author, without undue reservation.

## Ethics Statement

Ethical review and approval was not required for the study on human participants in accordance with the local legislation and institutional requirements. The patients/participants provided their written informed consent to participate in this study.

## Author Contributions

The author confirms being the sole contributor of this work and has approved it for publication.

## Conflict of Interest

The author declares that the research was conducted in the absence of any commercial or financial relationships that could be construed as a potential conflict of interest.
